# A conceptual framework to address administrative and infection control barriers for animal-assisted intervention programs in healthcare facilities: Perspectives from a qualitative study

**DOI:** 10.1017/ice.2021.24

**Published:** 2021-01-25

**Authors:** Kathryn R. Dalton, Peter Campbell, William Altekruse, Roland J. Thorpe, Jacqueline Agnew, Kathy Ruble, Karen C. Carroll, Meghan F. Davis

**Affiliations:** 1 Department of Environmental Health and Engineering, Johns Hopkins University Bloomberg School of Public Health, Baltimore, Maryland; 2 School of Medicine, University of Maryland, Baltimore, Maryland; 3 School of Social Work, University of Maryland, Baltimore, Maryland; 4 Department of Health, Behavior and Society, Johns Hopkins University Bloomberg School of Public Health, Baltimore, Maryland; 5 Department of Pediatric Oncology, Johns Hopkins University School of Medicine, Baltimore Maryland; 6 Division of Medical Microbiology, Department of Pathology, Johns Hopkins University School of Medicine, Baltimore, Maryland; 7 Department of Molecular and Comparative Pathobiology, Johns Hopkins University School of Medicine, Baltimore, Maryland

*To the Editor*—Animal-assisted intervention (AAI) programs, used extensively in healthcare facilities, have numerous reported benefits to patients.^1–3^ These programs have increasingly been used for healthcare workers, as a targeted intervention to reduce occupational stress and burnout symptoms.^4^ However, barriers, specifically infection control concerns, prevent AAI programs from being used in many hospitals and among their diverse populations. This has become more apparent during the coronavirus disease 2019 (COVID-19) pandemic, and many AAI programs have been suspended due to apprehension about coronavirus spread, despite the critical need for proven mental health support programs for patients and employees during this taxing period.

This qualitative study aimed to capture opinions pertaining to benefits and concerns related to AAI from individuals directly involved in hospital programs, particularly occupational health benefits for hospital staff and infectious disease concerns. We report on these key stakeholders’ perspectives and experiences and, through these reports, present a conceptual framework to recommend measures to better implement and support these programs. Although we focused our research on infectious diseases broadly, participant responses and our research findings are reflective and applicable to concerns for AAI programs related to the COVID-19 pandemic.

As part of a larger study on hospital AAI program-related risks and exposures, we interviewed 37 healthcare workers and therapy animal handlers from multiple hospitals. We thematically coded interview transcriptions based on deductive programmatic framework analysis. The study underwent research ethics review and approval. Further details on methodology and study participants have been previously published.^5^

Participants reported that these programs did benefit hospital staff by reducing stress and bolstering morale. They felt this led to an improvement in job performance through increased employee engagement, and by providing an “additional tool in their toolbox” for improved patient care. Finally, these programs were reported to be a gateway to other therapy programs, such as mental health counseling. In spite of these cited benefits, participants identified administrative barriers to implementation, such as balancing clinical duties. They conveyed that these obstacles could be overcome with appropriate leadership, and from collaboration across the hospital and management “buy-in,” to underscore the value of staff inclusion in AAI.

Infection concerns were reported as a frequent barrier to program implementation, both for patient and healthcare worker use. Participants described their concern of the dog serving as an intermediary vector of pathogen spread among patients, staff, and the hospital environment. However, many participants, both pet therapy handlers and healthcare workers, felt this risk was minimal due to effective control measures, which should target the animal, the patients, and the hospital environment, designed with practical input from multiple stakeholders. The primary facilitator to appropriately enact control measures was the designation of individuals responsible for safety, and relevant training for all individuals involved with these programs about potential infectious risks and mitigation strategies.

**Figure 1.**
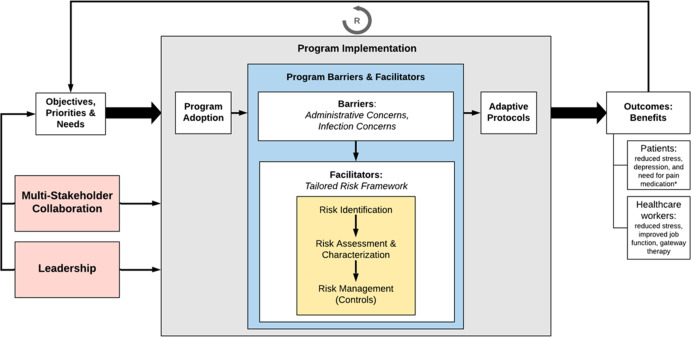
Conceptual Framework for Hospital Animal-Assisted Intervention Program Implementation Adapted from CFIR and EPA Risk Framework (yellow box). Blue box = program barriers and facilitators, grey box = program implementation, red boxes = external influences. Circled arrow with R = positive reinforcing feedback loop, where appropriate program implementation leads to an increase in program benefits, which validates and increases hospital needs for these programs. * Most commonly documented patient benefits from systematic reviews of previous literature (Bert et al., 2016; Kamioka et al., 2014; Waite et al., 2018)

Based on these reports, we developed a conceptual framework (Fig. 1), adapted from the Consolidated Framework for Implementation Research^6^ and the Environmental Protection Agency’s Risk Management Framework,^7^ which links our major themes in the context of program implementation. Hospital objectives and needs feed into program implementation, accomplished by addressing program barriers through facilitators (blue box). Perceived barriers, both administrative and infection risk as described, can be addressed through a risk management framework (yellow box): (1) identify the hazard (eg, infection concerns), (2) assess and characterize said hazard, and (3) hazard management through applying and monitoring control measures. This approach results in an adaptive protocol based on individual program needs. Critical to the design and execution of program implementation is multiple stakeholder and hospital leadership engagement (red boxes) to ensure diverse, comprehensive input on protocols. Implementing adaptive AAI programs, through targeted facilitators, results in program benefits for both patients and staff, such as those listed in the figure, since many program barriers and facilitators apply to both. This ultimately creates a reinforcing feedback loop improving program implementation by substantiating hospital needs.

Our qualitative study provided insight into appropriate AAI program implementation, both directed towards patients and HCW, based on the unique experiences and perspectives from individuals actively involved in these programs with crucial roles in their administration. Through participant reports and developing our conceptual framework, we identified 3 major areas for program improvement. First is the need for a tailored risk assessment to understand barriers unique to individual programs, hospitals, departments, and patient populations, to develop adaptive protocols. Secondly, leadership roles, or “champions,” are essential to advocate for the programs’ worth, plus communicate and ensure adherence to policies critical to success. Lastly, collaboration across the hospital is needed to design protocols for AAI with input from multiple stakeholder groups to ensure that program guidelines are comprehensive and practical.

This conceptual framework can serve as a scaffold for hospitals wishing to start or extend AAI programs, and it is noteworthy for hospital administrators, healthcare epidemiologists, and occupational health specialists. More currently, this framework can be used to design plans to restart suspended AAI programs due to COVID-19, as well as potentially other patient well-being volunteer programs. The detailed level of contextual qualitative data obtained from our participants can be utilized to develop a practical quantitative survey to collect data from a wider scope of hospitals and participant groups to increase our recommendations’ generalizability. The results of this, and future work, will have significant implications in the utilization and preservation of these valuable AAI programs.
